# Multiple cross displacement amplification combined with nanoparticle-based lateral flow biosensor for rapid and sensitive detection of Epstein-Barr virus

**DOI:** 10.3389/fcimb.2023.1321394

**Published:** 2024-01-08

**Authors:** Xinbei Jia, Juan Zhou, Fei Xiao, Xiaolan Huang, Wenqiang He, Wen Hu, Yaru Kong, Weiheng Yan, Jie Ji, Yuwei Qi, Yi Wang, Jun Tai

**Affiliations:** ^1^ Department of Otorhinolaryngology Head and Neck Surgery, Children’s Hospital Capital Institute of Pediatrics, Chinese Academy of Medical Sciences & Peking Union Medical College, Beijing, China; ^2^ Experimental Research Center, Capital Institute of Pediatrics, Beijing, China; ^3^ Department of Otolaryngology, Head and Neck Surgery, Children’s Hospital Capital Institute of Pediatrics, Beijing, China; ^4^ Department of Otorhinolaryngology Head and Neck Surgery, Beijing Children′s Hospital, Capital Medical University, National Center for Children′s Health, Beijing, China

**Keywords:** Epstein-Barr virus, multiple cross displacement amplification, lateral flow biosensor, MCDA-LFB, nasopharyngeal carcinoma

## Abstract

**Introduction:**

Epstein-Barr virus (EBV) is a highly dangerous virus that is globally prevalent and closely linked to the development of nasopharyngeal cancer (NPC). Plasma EBV DNA analysis is an effective strategy for early detection, prognostication and monitoring of treatment response of NPC.

**Methods:**

Here, we present a novel molecular diagnostic technique termed EBV-MCDA-LFB, which integrates multiple cross displacement amplification (MCDA) with nanoparticle-based lateral flow (LFB) to enable simple, rapid and specific detection of EBV. In the EBV-MCDA-LFB system, a set of 10 primers was designed for rapidly amplifying the highly conserved tandem repeat BamHI-W region of the EBV genome. Subsequently, the LFB facilitate direct assay reading, eliminating the use of extra instruments and reagents.

**Results:**

The outcomes showed that the 65°C within 40 minutes was the optimal reaction setting for the EBV-MCDA system. The sensitivity of EBV-MCDA-LFB assay reached 7 copies per reaction when using EBV recombinant plasmid, and it showed 100% specificity without any cross-reactivity with other pathogens. The feasibility of the EBV-MCDA-LFB method for EBV detection was successfully validated by 49 clinical plasma samples. The complete detection process, consisting of rapid template extraction (15 minutes), MCDA reaction (65°C for 40 minutes), and LFB result reading (2 minutes), can be finalized within a 60-minutes duration.

**Discussion:**

EBV-MCDA-LFB assay designed here is a fast, extremely sensitive and specific technique for detecting EBV in field and at the point-of-care (PoC), which is especially beneficial for countries and regions with a high prevalence of the disease and limited economic resources.

## Introduction

Epstein-Barr virus (EBV) is a human B lymphotropic gamma-herpes virus that is predominantly transmitted via respiratory secretions. This virus can persist in individuals throughout their lifespan following the initial infection, and over 90% of the global population exhibited a positive serological reaction (Cohen, 2000; Nowalk and Green, 2016; Kerr, 2019). Nasopharyngeal carcinoma (NPC), which has a close etiological association with EBV, is a malignant epithelial tumor that is highly prevalent in southern China, Southeast Asia, and North Africa. (Tan et al., 2020b). In endemic areas, more than 97% of NPC cases are positive for EBV, with males having an incidence rate of up to 30.9 per 100,000 persons per year (Cohen et al., 2011; Li et al., 2014). The detection of EBV DNA in plasma samples provides a valuable and cost-effective method for early screening and monitoring of asymptomatic NPC cases, which enables prompt treatment, reduces mortality and morbidity, and avoids the adverse effects and financial burden associated with extensive radiation and chemotherapy. Therefore, it is imperative to have reliable EBV DNA detection in order to perform early screening for NPC risk groups (Chan et al., 2017; Tan et al., 2020a)

Various detection methods can be utilized for the diagnosis of EBV infection. Serological assays, such as enzyme immunoassay, immunofluorescence assay, and chemiluminescent immunoassay, are the preferred diagnostic tools for detecting EBV-specific antibodies at different infection stages. The combination of three analytes including IgM antibodies against the viral capsid antigen (VCA), IgG antiVCA antibodies, and IgG antibodies toward EBV nuclear antigen-1 (EBNA-1), supplemented by IgG antibodies to early antigen (EA), is extensively utilized to differentiate between acute, past, or reactivated EBV infection (Crowley et al., 2012; Corrales et al., 2014; Niller and Bauer, 2017). However, these methods merely serve as indicators of viral infection and do not directly reflect *in vivo* EBV replication (Corrales et al., 2014). In tumor biopsy samples, the gold standard technique for detecting latent EBV is *in situ* hybridization (ISH) using DNA or RNA probes specific to EBV-encoded RNA (EBERs) (Fanaian et al., 2009; AbuSalah et al., 2020). Given the abundant expression (in millions of copies) of EBERs in latently-infected cells, they serve as dependable molecular markers crucial for detecting and localizing EBV-infected tissue samples (AbuSalah et al., 2020; Tonoyan et al., 2020). Nevertheless, the drawbacks of complex, tedious, and time-consuming procedures with a high possibility of obtaining false negative results restrict its extensive utilization in clinical diagnosis (AbuSalah et al., 2020). Real-time quantitative PCR (qPCR) is the main molecular biology technique for contemporary EBV viral quantification. This method facilitates the real-time monitoring and quantification of amplified targeted nucleic acid sequences by employing either fluorescent probes or an intercalating dye, based on amplifying a conserved sequence. (AbuSalah et al., 2020; Tonoyan et al., 2020). Although highly sensitive, specific, and efficient, PCR-based techniques require complex thermal cycling apparatus for target DNA amplification and need gel electrophoresis or probe hybridization techniques for result analysis, hindering its widespread use in resource-limited regions (Wang et al., 2016a; Wang et al., 2016b; Yuan et al., 2020). Hence, there is a compelling need to establish advanced detection methods for the diagnosis of EBV infection.

Taking into account the aforementioned challenges, Wang et al. (Wang et al., 2015) devised a novel isothermal nucleic acid amplification technique called multiple cross displacement amplification (MCDA). While the MCDA assay may not quantity the *in vivo* replication of EBV copies as efficiently as the qPCR technique, it remains extensively adopted for detecting various pathogens in clinical, biological, and environmental samples. This popularity arises from its distinctive advantages, including rapidity, cost-effectiveness, repeatability, as well as high specificity and sensitivity, as demonstrated in various studies (Wang et al., 2016a; Cao et al., 2021; Cheng et al., 2023; Zhou et al., 2023). In the MCDA system, only a single DNA polymerase with strand displacement activity is employed to amplify the target templates at a constant temperature. In particular, the MCDA utilizes a set of 10 primers, including 2 cross primers (CP1 and CP2), 2 displacement primers (F1 and F2), and 6 amplification primers (D1, C1, R1, D2, C2, and R2), to recognize the target sequence, thereby ensuring assay’s specificity. A comprehensive explanation of the MCDA technique can be found in a prior study (Wang et al., 2015).

The practical use and commercial value of MCDA technique greatly hinge on the availability of a simple and rapid method for indicating its results. Nanoparticle-based lateral flow biosensors (LFB) are an excellent choice for monitoring MCDA reaction, because LFB is easy to construct and operate, cost-effective and user-friendly (requiring no training), providing rapid results visible to the naked eye within 2 minutes, as well as having the potential for point-of-care (POC) diagnosis (Quesada-González and Merkoçi, 2015; Chen et al., 2021). By capitalizing on these advantages, various LFBs have been successfully designed for coupling with MCDA assay (MCDA-LFB), which have been widely applied for diagnosing various infections, including COVID-19, Tuberculosis and monkeypox et al. (Li et al., 2020; Huang et al., 2021; Zhou et al., 2023).

**Figure 1:**
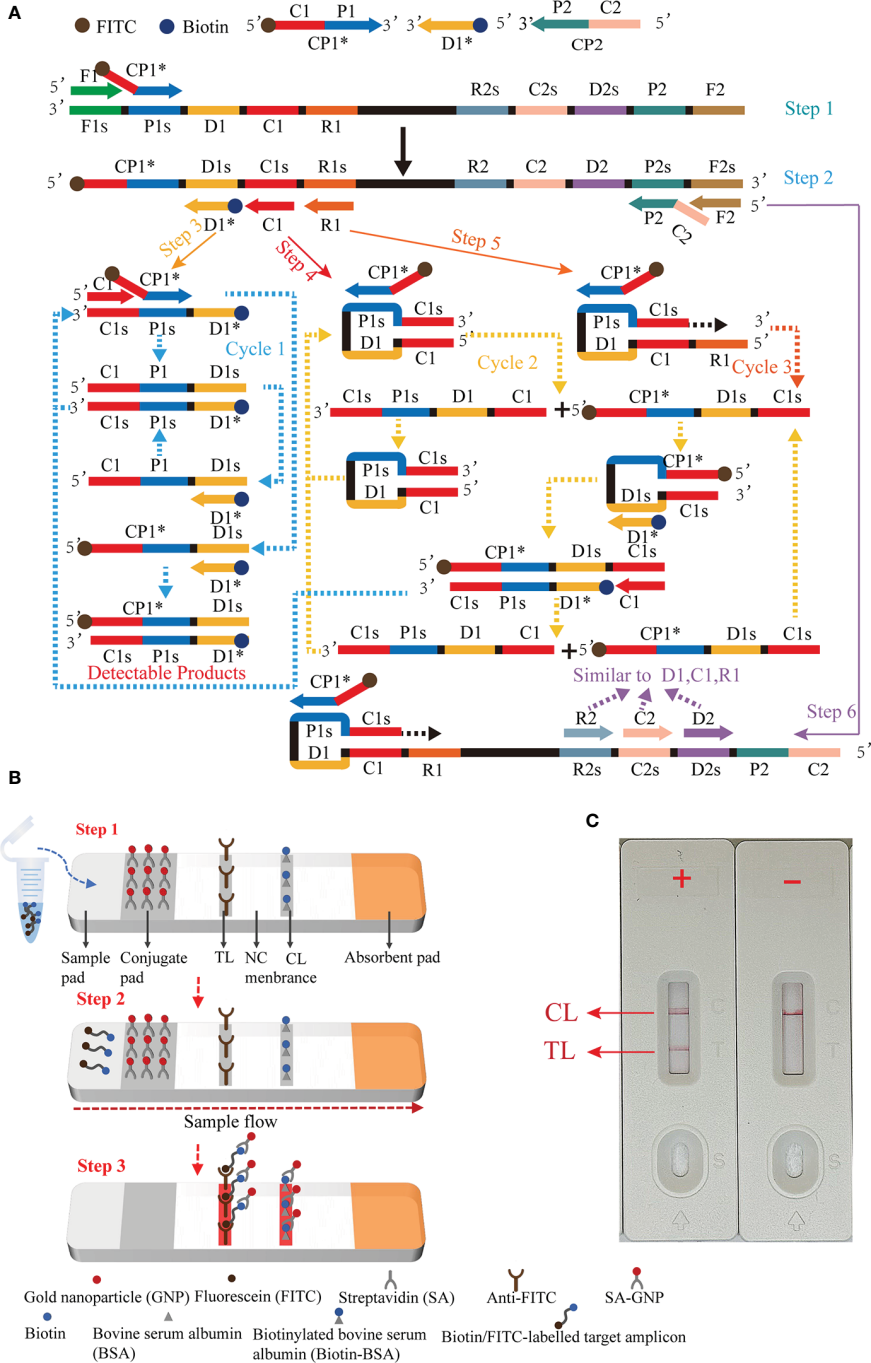
Outline of the multiple cross displacement amplification combined with lateral flow biosensor. **(A)** The principle of multiple cross displacement amplification. **(B)** The schematic diagram of the LFB for visualized analysis of EBV-MCDA. The aliquot of MCDA amplicons and running buffer (50 µl) were added to the sample region (step 1). The running buffer containing amplification products flowed along the LFB, and the immobilized streptavidin (SA) coated-gold nanoparticle (GNP) was rehydrated in the conjugate region (step 2). In the positive sample, the biotin/FITC-labelled amplicons combined with SA-GNP were specifically captured by immobilized anti-FITC for visualization readout at the test line. In order to ensure the proper function of the strip, the extra SA-GNP was subsequently seized by biotinylated bovine serum albumin (Biotin-BSA) for visualization at the control line (step 3). **(C)** Interpretation of the EBV-MCDA-LFB assay results. For positive outcomes, two red lines appeared on the TL and CL regions. For negative outcomes, only one red line appeared on the CL region. CP1*, 5'-labeled with FITC; D1*, 5'-labeled with biotin.

Here, we present a molecular detection technique that combines MCDA with LFB for rapid, simple, and sensitive specific detection of EBV, termed EBV-MCDA-LFB. The principle of the EBV-MCDA-LFB assay is depicted in **Figure 1** and its feasibility was successfully validated using suspected EBV-positive clinical plasma samples.

## Materials and methods

### Reagents and instruments

Visual detection reagent (VDR), LFB, and DNA isothermal amplification kits were purchased from HuiDeXin Biotic Co., Ltd (Tianjin, China). Viral DNA purification kits were supplied by TransGen Biotic Co., Ltd (Beijing, China). The oligomers were synthesized and purified at a high liquid chromatography purification grade by DIA-UP BIOTECH Co., Ltd (Beijing, China). Commercially constructed plasmids and the labeled primers were provided by TianYi-Huiyan Biotech. Co., Ltd. (Beijing, China). Commercial PCR diagnosis kits for EBV were purchased from Sansure Biotic Co., Ltd (Changsha, China). The kinetic turbidity curves were plotted using Loopamp Real-Time Turbidimeter (LA-320C) produced by Eiken Chemical Co., Ltd. (Tokyo, Japan).

### Preparation of pathogens and clinical samples

A total of 49 plasma samples were collected from patients with suspected EBV infection in Children’s Hospital Capital Institute of Pediatrics from April 2023 to August 2023. Genomic DNA of the samples were extracted using the commercially available kit (Viral DNA purification kits, TransGen) as per the manufacture’s instruction, which was a solid-phase extraction method that largely applied in point of care (POC) settings. The extract nucleic acid was stored at -20°C for subsequent use. Moreover, 21 non-EBV isolated pathogens obtained from the Chinese Center for Disease Control and Prevention (CDC) were used to validate the specificity of the EBV-MCDA-LFB (**Table 1**).

**Table 1:** Pathogens used in the Analytical Specificity identification.

No.	pathogen	No. of strains	Source of strain^a^	EBV-MCDA-LFB Result^b^
1	EBV (recombinant plasmid)	1	_	P
2	EBV (clinical sample)	1	CIP	P
3	Respiratory syncytial virus	1	Isolated strains (ICDC)	N
4	Human rhinovirus	1	Isolated strains (ICDC)	N
5	Rubella virus	1	Isolated strains (ICDC)	N
6	Influenza virus B	1	Isolated strains (ICDC)	N
7	Adenovirus (ADV9)	1	Isolated strains (ICDC)	N
8	Measles virus	1	Isolated strains (ICDC)	N
9	Herpes simplex virus	1	Isolated strains (ICDC)	N
10	Coxsackievirus	1	Isolated strains (ICDC)	N
11	Parainfluenza virus (PIV1)	1	Isolated strains (ICDC)	N
12	Parainfluenza virus (PIV3)	1	Isolated strains (ICDC)	N
13	Vesicular stomatitis virus	1	Isolated strains (ICDC)	N
14	Sendai virus	1	Isolated strains (ICDC)	N
15	Dengue virus	1	Isolated strains (ICDC)	N
16	Visna virus	1	Isolated strains (ICDC)	N
17	*Haemophilus influenzae*	1	Isolated strains (ICDC)	N
18	*Neisseria meningitidis*	1	Isolated strains (ICDC)	N
19	*Neisseria lactate*	1	Isolated strains (ICDC)	N
20	*Streptococcus pneumoniae*	1	Isolated strains (ICDC)	N
21	*Staphylococcus aureus*	1	Isolated strains (ICDC)	N
22	*Mycobacterium tuberculosis*	1	Isolated strains (ICDC)	N
23	*Klebsiella pneumoniae*	1	Isolated strains (ICDC)	N

a CIP, Children’s Hospital Capital Institute of Pediatrics; ICDC, National Institute for Communicable Disease Control and Prevention, Chinese Center for Disease Control and Prevention.

b P, Positive; N, Negative.

### Standard plasmid construction and EBV-MCDA primer design

**Figure 2:**
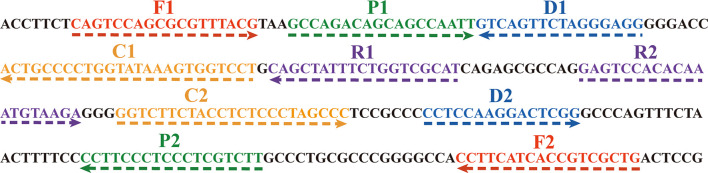
The sequences of the BamHI-W gene were used to design MCDA primers. The right arrows and left arrows are sense and complementary DNA sequences which were used in this study, respectively.

In the MCDA-based assay for target DNA detection, the BamHI-W fragment (Genebank Accession: NC_001345.1: 21217-24288), which is a highly conserved tandem reiterate sequence from the EBV genome, was cloned into the customized pUC57 vector (Sanosyan et al., 2017). According to the principle of MCDA technique (Wang et al., 2015), a set of 10 primers targeting ten distinct regions of target sequence was designed using the PrimerExplorer V4 (Eiken Chemical, Japan) and PREMIER 5.0. Specificity of the primers was determined using NCBI Primer-BLAST (Basic Local Alignment Search Tool). Moreover, the primers CP1 and D1 were further modified by labeling FITC and biotin at the 5’ end, respectively, for LFB detection. The details of primer design, primer, locations, sequences, and alterations are shown in **Figure 2**; **Table 2**.

**Table 2:** Primers used for multiple cross displacement amplification.

Primers^a^	Sequence (5’-3’)^b^	Length^c^
F1	CAGTCCAGCGCGTTTACG	18 nt
F2	CAGCGACGGTGATGAAGG	18 nt
CP1	AGGACCACTTTATACCAGGGGCAGT-GCCAGACAGCAGCCAATT	43 mer
CP1*	FITC-AGGACCACTTTATACCAGGGGCAGT-GCCAGACAGCAGCCAATT	43 mer
CP2	GGTCTTCTACCTCTCCCTAGCCC-AAGACGAGGGAGGGAAGG	41 mer
C1	AGGACCACTTTATACCAGGGGCAGT	25 nt
D1	CCTCCCTAGAACTGAC	16 nt
D1*	Biotin-CCTCCCTAGAACTGAC	16 nt
R1	ATGCGACCAGAAATAGCTG	19 nt
C2	GGTCTTCTACCTCTCCCTAGCCC	23 nt
D2	CCTCCAAGGACTCGG	15 nt
R2	GAGTCCACACAAATGTAAGA	20 nt

a CP1*, 5’-labeled with FITC when used in MCDA-LFB assay; D1*, 5’-labeled with biotin when used in MCDA-LFB assay.

b FITC, fluorescein isothiocyanate.

c nt, nucleotide; mer, monomeric unit

### The standard MCDA assay

The EBV-MCDA reaction was conducted in a total of 25 µl amplification mixture containing 0.4 µM each of displacement primer (F1 and F2), 0.8 µM each of amplification primer (C1, C2, R1, R2, D1∗, and D2), 1.2 µM each of cross primer (CP1∗and CP2), 12.5 µl 2× reaction mixture, 1.5 µl VDR, 1 µl *Bst* 2.0 DNA polymerase, and 1 µl DNA template from standard plasmid or 5 µl from clinical specimens, and the mixture volume add up to 25 µl with double distilled water. Then, the MCDA reaction mixtures were incubated at 64°C for 40 minutes and 80°C for 5 minutes to stop the amplification. To examine the feasibility of the EBV-MCDA primers, amplification mixtures containing the 1 µl non-EBV pathogens (herpes simplex virus [HSV] and respiratory syncytial virus [RSV]) were utilized as negative controls, and mixtures containing double distilled water were used as blank control.

### Introduction of nanoparticle-based lateral flow biosensor

The schematic diagram of the LFB for visualized analysis of EBV-MCDA amplicons was illustrated in **Figure 1B**. This biosensor comprised four components: a sample pad, a conjugate pad, an NC membrane (nitrocellulose membrane), and an absorption pad, all adhering to a plastic adhesive backing pad. Particularly, the sample pad was the place where analytes and running buffer were dropped; the conjugate pad was coated with streptavidin-gold nanoparticles (SA-GNPs, in 0.01 M phosphate buffered saline (PBS, PH 7.4), which could be rehydrated by the running buffer and further combined with analyte labeled with biotin; the NC membrane was coated with two capture regents, i.e., anti-FITC (0.15 mg/ml) and biotin-BSA (4 mg/ml), which were applied to the capture FITC-labeled and streptavidin-containing analytes, respectively; the absorption pad was utilized to boot sample flow through the capillary migration force (Wang et al., 2016a). During monitoring, the amplified products and running buffer (0.01 M PBS, PH 7.4 with 1% Tween 20) were sequentially applied to the sample pad. Through capillary action, sample solution automatically flowed along the LFB from the sample pad to the absorbent pad, rehydrating the SA-GNPs in the conjugate region. The target analyte induced the aggregation of reporter molecules at the test zone, resulting in the emergence of a visible line on the test zone.

### EBV-MCDA products detection

The current study employed three monitoring techniques, including a turbidimeter (Loopamp RealTime Turbidimeter, LA-320C), visual detection reagents, and lateral flow biosensors to analyze EBV-MCDA products. The real-time turbidimeter measured turbidity resulting from the accumulation of magnesium pyrophosphate, a white precipitate formed during the MCDA reaction. A reaction was deemed positive when the turbidity value greater than 0.1. The visual indicator regent worked as an indicator of nucleic acid amplification. At the initial stage of amplification, the reaction mixtures were colorless due to the VDR degraded to A and B groups with high temperature; once the double-strand nucleic acids were generated, they will combine with the A group and the reaction mixtures will display green in color, while the ones absence of double-strand nucleic acid were still colorless. In the LFB, both CL and TL exhibited a red line indicating a positive amplification reaction, whereas only CL showed a single red line indicating a negative amplification reaction.

### Optimal temperature of the EBV-MCDA assay

Optimizing the reaction temperature is crucial for enhancing the amplification efficiency of the MCDA reaction. Here, the EBV-MCDA reactions were carried out at eight constant temperatures ranging from 61 to 68°C with a 1°C interval for 40 minutes. HSV was used as negative control (NC), and double-distilled water (DW) was used as a blank control. The real-time turbidimeter was used to analyzed the reaction products.

### Sensitivity of the EBV-MCDA-LFB assay

10-fold serial dilutions of the standard plasmids (7.0 x 10^4^ to 7.0 x 10^-2^ copies per microliter) were prepared to confirm the analytical sensitivity and limit of detection (LoD) of the MCDA reaction for EBV detection. The products were then detected by VDR, turbidimeter, and LFB methods. Each dilution was tested at least three times to assess the analytical sensitivity.

### Optimal isothermal amplification time of EBV-MCDA assay

After determining the LoD and the optimal reaction temperature, the plasmid concentration corresponding to the LoD level was selected to determine the optimal amplification time. Four different reaction times (10, 20, 30, and 40 minutes) were compared under the optimized EBV-MCDA reaction conditions. The amplification results were interpreted using VDR and LFB methods at each time point.

### Optimizing amplicon content for LFB assay

The optimal amplicon content for the LFB assay was determined and validated by employing varied volumes (0.5, 1, 1.5 µl) of EBV-MCDA products. Under the previously optimized conditions, the amplification reactions were conducted with 1 µl of plasmid concentration, corresponding to the LoD level, as the template, while double-distilled water served as the negative control. After amplification, three different volumes of the amplified products (0.5, 1, 1.5 µl) were extracted via a pipette gun and subsequently applied to the LFBs. Subsequently, following a 2-minute flow at room temperature, the results were observed and visually recorded without any additional aids.

### Specificity of the EBV-MCDA-LFB assay

The analytical specificity of the EBV-MCDA-LFB assay was evaluated using seven bacterial and 14 viral genomic DNA templates (**Table 1**) under the conditions mentioned above. The products were tested using VDR and LFB. Each sample was tested independently in at least three experiments.

### Examination of the EBV-MCDA-LFB assay using clinical samples

To further validate the feasibility of the EBV-MCDA-LFB technology established in this study for clinical EBV detection, 49 plasma samples were collected from patients with suspected EBV infection at Children’s Hospital Capital Institute of Pediatrics. These samples were detected by real-time quantitative PCR (qPCR) and EBV-MCDA-LFB, respectively, and the detection results of the two methods were compared. Following the manufacturer’s instructions, the real-time qPCR processes were carried out in a volume of 50 µl, consisting of 10 µl of extracted DNA template and 40 µl of PCR mixture containing primers, probes, dNTPs, Mg^2+^, Taq polymerase, UDG enzyme, and the plasmid of target gene fragment. PCR amplification was performed for 45 cycles, including denaturation at 94°C for 15s, annealing and extension at 57°C for 30s. The amplification products were analyzed using the 7500 real-time PCR technology (Applied Biosystems, United States). The EBV-MCDA-LFB assay was conducted under the optimized reaction condition as described previously.

## Results

### Overview of the MCDA-LFB assay

Principle of MCDA-LFB assay for EBV detection was illustrated in **Figure 1**. Briefly, EBV was detected by amplifying the target sequence using MCDA technique (**Figure 1A**) and interpreting the results using LFB platform (**Figure 1B**). The MCDA reaction was performed as previously described (Wang et al., 2015), which only employed a DNA polymerase possessing strand displacement activity and a set of 10 specifically designed primers covering 10 distinct regions of target sequence. Particularly, the conventional primers CP1 and D1 were labeled with different haptens (FITC and biotin) in this study in order to enable LFB detection (termed CP1* and D1*). During MCDA reaction stage (**Figure 1A**), the primer CP1* initiated the amplification reaction, then the generated single-stranded DNA was released by the strand displacement DNA synthesis primed by F1 (step 1). The CP1* primed single-stranded DNA would then be employed as template for DNA synthesis primed by primers C1, D1*, R1 and the reverse primers CP2 and F2 (step 2). In the subsequent cycling, various amplicons will be generated, including the double labeled amplicons primed by CP1*/D1* (steps 3-6) and several stem-loop DNA. The cycling reaction will yield plenty of products comparable to PCR reaction within less than one hour. Thus, adequate amount of CP1*/D1*-amplicons will be detected and visualized by LFB platform.

The schematic diagram of the LFB for visualized analysis of EBV-MCDA amplicons was illustrated in **Figure 1B**. After successfully amplifying the template DNA, an optimal quantity of MCDA amplicons aliquot along with 50 µl of running buffer were sequentially added to the sample region of the biosensor (**Figure 1B**, step 1). Then, the capillary action facilitated the flow of the running buffer, containing the amplification product, along the LFB, leading to the rehydration of the streptavidin-GNP in the conjugate region (**Figure 1B**, step 2). The biotin/FITC-labelled target amplicons interacted with SA-GNP and were specifically captured by immobilized anti-FITC, eventually appearing on the TL, indicating a positive result. Additionally, the functionality of the biosensor was ensured by the capture of excess SA-GNPs by biotinylated bovine serum albumin immobilized on the CL (**Figure 1B**, step 3).

### Confirmation and detection of EBV-MCDA-LFB products

**Figure 3:**
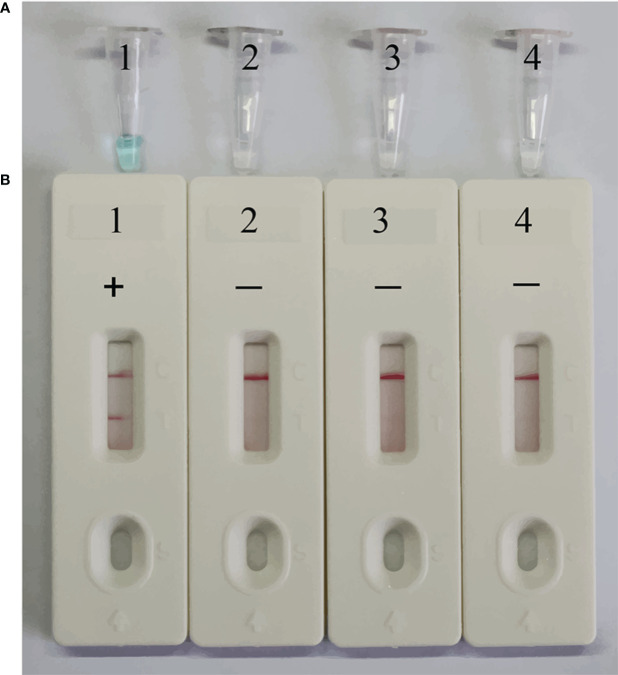
Confirmation of EBV-MCDA-LFB assay for EBV detection. Amplification products were visually analyzed by VDR **(A)** and LFB **(B)**. Tube 1/biosensor 1, positive amplification of EBV standard plasmid; tube 2/biosensor 2, negative control of viral DNA of HSV; tube 3/biosensor 3, negative control of viral RNA of RSV; tube 4/biosensor 4, blank control of double distilled water (DW).

To confirm the feasibility of the selected primers in the MCDA assay, EBV standard plasmid, viral DNA of HSV, and viral RNA of RSV were employed as templates for the MCDA assay at 64°C for 40 minutes. VDR and LFB monitoring techniques were utilized to interpret the EBV-MCDA results. The outcomes revealed that positive result was observed only in tube containing EBV standard plasmid templates, while negative results were shown in the HSV, RSV, and blank control groups (**Figures 3A, B**). These findings confirmed the validity of the EBV-MCDA primers utilized in the study for detecting EBV through MCDA-LFB.

### The optimal reaction temperature for MCDA assay

**Figure 4:**
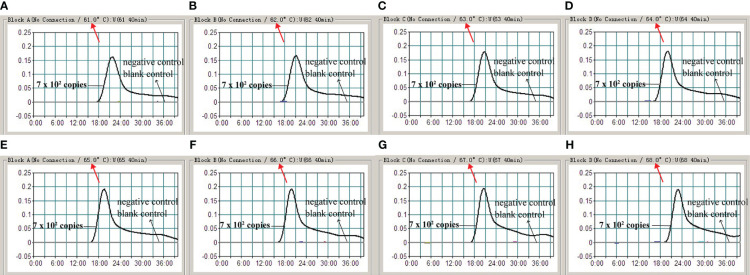
The optimal reaction temperature for MCDA assay. The standard MCDA reactions for the detection of EBV were monitored by real-time measurement of turbidity. The corresponding curves of concentrations of DNA were marked in the figures. The threshold value was 0.1 and the turbidity value of >0.1 was considered as positive outcome. Eight kinetic curves **(A-H)** were plotted at continuously varying temperatures (61-68°C, 1°C intervals) with the EBV recombinant plasmid at the level of 7.0 x 10^2^ copies per reaction.

To confirm the optimal temperature for primer-template binding and polymerase extension in the EBV-MCDA assay, the amplification temperatures were regulated within the range of 61 to 68°C at 1°C intervals. EBV standard plasmids were used as templates at a concentration of 7.0 x 10^2^ copies per microliter. The reactions were analyzed by real-time turbidimetry, and the kinetic curves corresponding to the eight temperatures were plotted. 65°C was finally determined to be the optimal reaction temperature for the EBV-MCDA system since this temperature can facilitate the faster achievement of the 0.1 turbidity threshold value (**Figure 4**). Therefore, all subsequent reactions were conducted at 65°C.

### Analytical sensitivity of the EBV- MCDA-LFB assay

**Figure 5:**
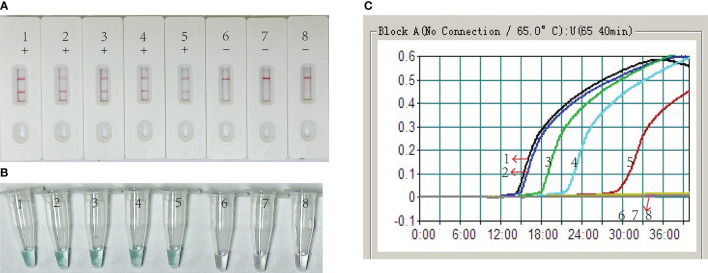
Analytical Sensitivity of EBV-MCDA-LFB using serial dilutions recombinant plasmid of the EBV. Three measurement techniques, including lateral flow biosensor **(A)**, visual detection reagent **(B)**, and real-time turbidity **(C)** were used to detect the amplification products. 1-8 represented the DNA levels of 7.0 x 10^4^, 7.0 x 10^3^, 7.0 x 10^2^, 7.0 x 10^1^, 7.0 x 10^0^, 7.0 x 10^-1^, 7.0 x 10^-2^ copies per microliter and blank control (DW), respectively.

Serial dilutions of EBV plasmids containing BamHI-W fragments (7.0 × 10^4^-7.0 × 10^-2^ copies per microliter) were used as templates to verify the analytical sensitivity of the MCDA-LFB assay for EBV detection. The MCDA reaction was performed under the optimal conditions determined above, and the results were evaluated through real-time turbidimeter, VDR, and LFB (**Figure 5**). According to the LFB biosensor, two visible red bands appeared in CL and TL regions at the plasmid concentrations of 7.0 × 10^4^ to 7 copies per microliter, indicating positive amplifications. Conversely, negative amplifications were observed at concentrations ranging from 7.0 × 10^-1^ to 7.0 x 10^-2^ copies per microliter and within the blank control group (**Figures 5A, B**). The typical kinetic plots from a real-time turbidimeter demonstrated the LoD level corresponding to a plasmid concentration of 7 copies per microliter (**Figure 5C**), which aligned with the outcomes obtained from VDR and LFB.

### Optimal reaction time of the EBV-MCDA-LFB assay

**Figure 6:**
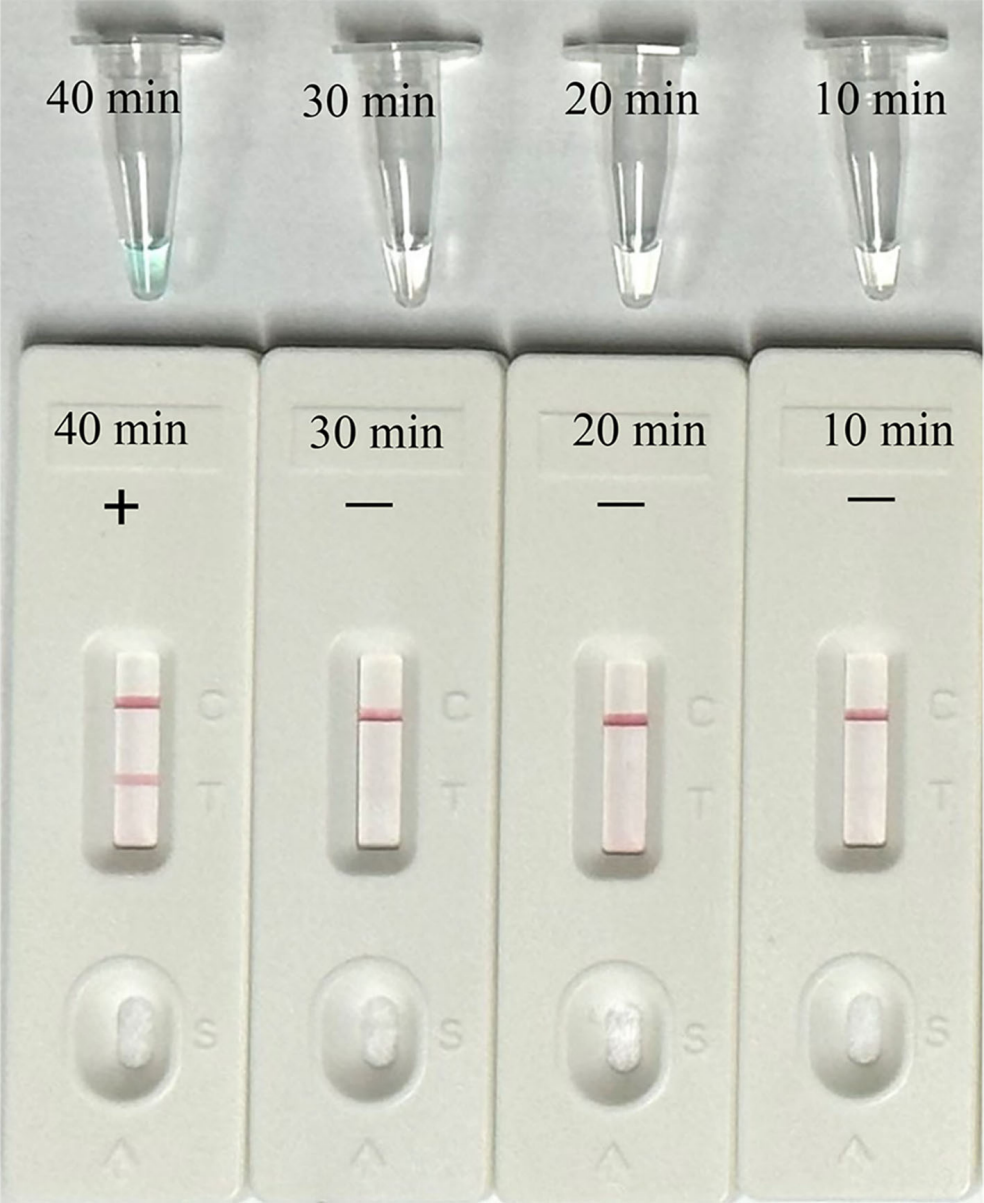
Optimal reaction time for EBV-MCDA-LFB Assay. Different reaction times (10, 20, 30, 40 min) were tested and compared at optimal amplification temperature (65°C) using the plasmid with a concentration of 7 copies per microliter.

Then, we identified the optimal duration time for the amplification stage of the EBV-MCDA-LFB assay. Four different reaction times (10, 20, 30, and 40 min) were examined and compared at 65°C using plasmid concentration of 7 copies per microliter as template. According to the results interpreted via VDR and LFB, the minimum time needed to detect the LoD level of EBV plasmid (7 copies per microliter) was 40 minutes (**Figure 6**). Thus, a 40-minute reaction time was determined to be the optimal duration for the EBV-MCDA-LFB assay.

### Optimizing amplicon content for LFB assay

**Figure 7:**
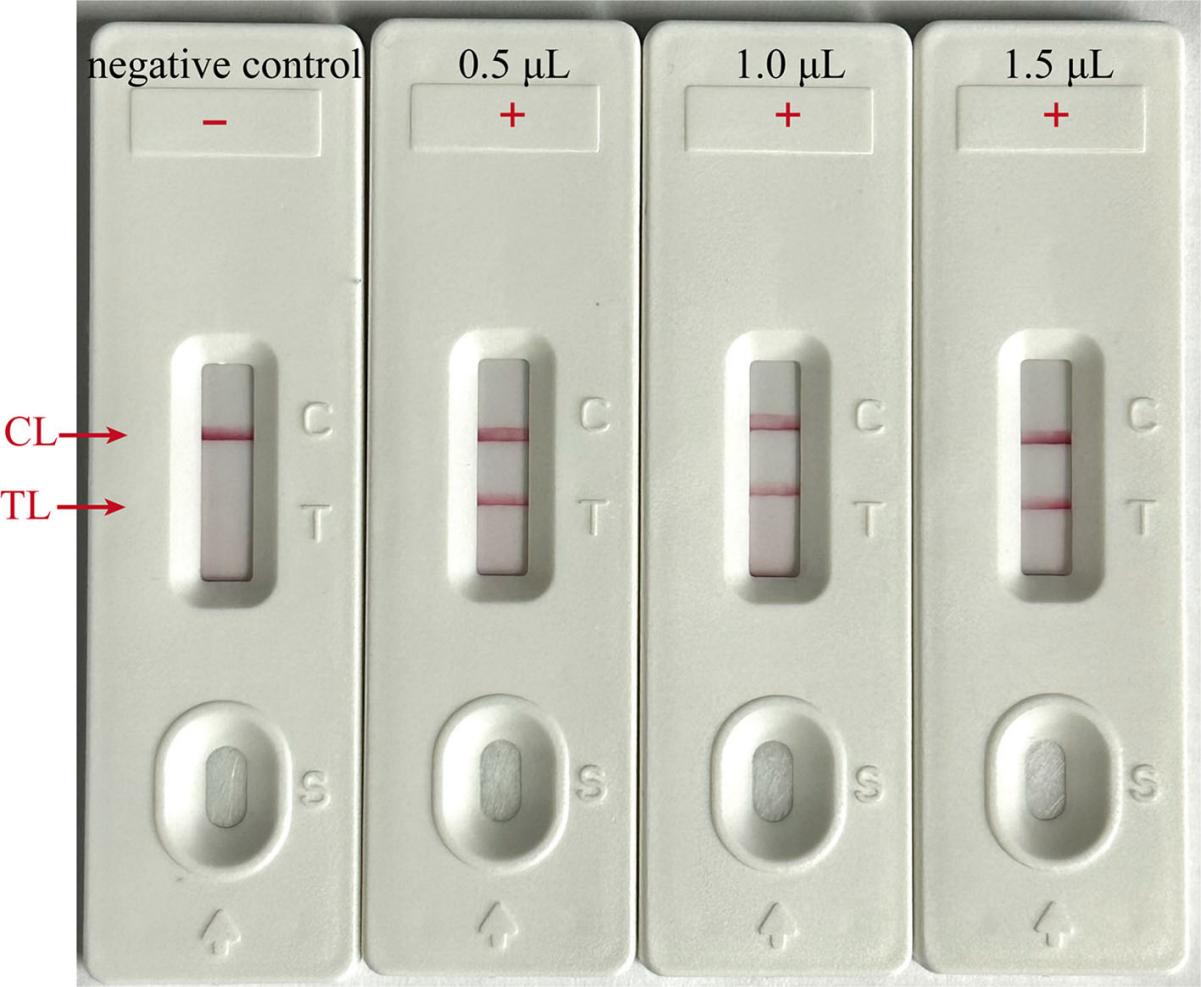
Optimal amplicon content for LFB Assay. Different volumes (0.5, 1 and 1.5 µl) of EBV-MCDA amplicon were extracted and dropped onto the LFBs to verify the sufficient content for effective detection by LFB assay; “ + “, positive; “-”, negative.

To ascertain the optimal amplicon amount for the LFB assay, the EBV-MCDA reaction was conducted using the plasmid with the concentration of 7 copies per microliter as template under the optimal reaction condition (65°C for 40 min), as determined previously. After the amplification reaction, three different volumes of the amplified products (0.5, 1, and 1.5 µl) were aspirated using a pipette gun and dropped onto the LFBs to observe the reactions on the CL and TL strips. As illustrated in **Figure 7**, all three LFBs, titrated with different volumes of amplified products, displayed two red lines at CL and TL within 2 minutes. In contrast, the negative control group exhibited only one red line at CL, indicating that a minimal volume of 0.5 µl of amplified product is adequate for effective detection by the LFB assay.

### Analytical specificity of the EBV-MCDA-LFB assay

**Figure 8:**
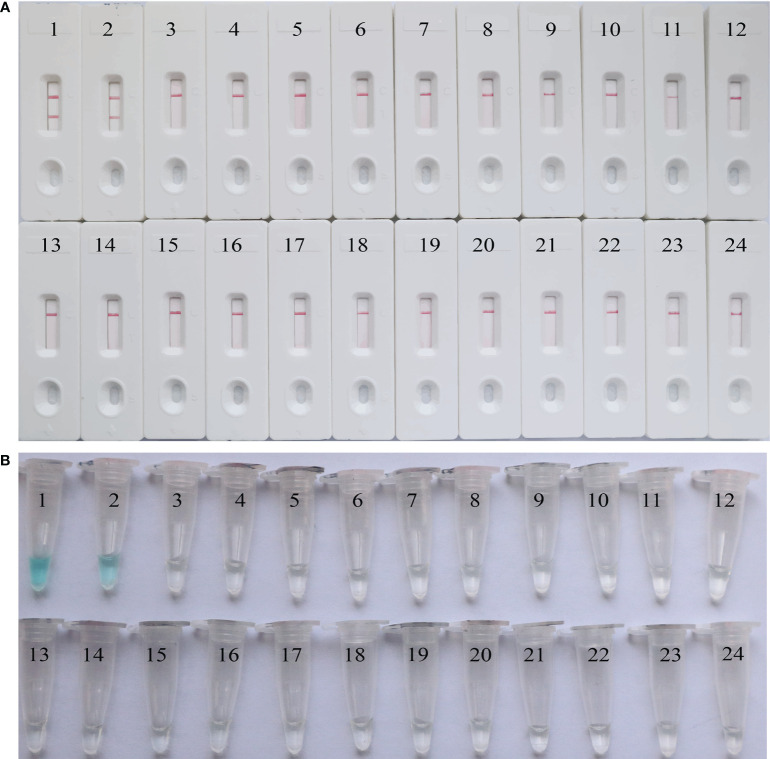
The analytical specificity of the EBV-MCDA-LFB assay. The specificity of the EBV-MCDA-LFB assay was evaluated using 21 non-EBV pathogens, and the results were monitored by means of LFB **(A)** and VDR **(B)**. 1, EBV standard plasmid; 2, EBV clinical sample; 3, Respiratory syncytial virus; 4, Human rhinovirus; 5, Rubella virus; 6, Influenza virus B; 7, Adenovirus (ADV9); 8, Measles virus; 9, Herpes simplex virus; 10, Coxsackievirus; 11, Parainfluenza virus (PIV1); 12, Parainfluenza virus (PIV3); 13, Vesicular stomatitis virus; 14, Sendai virus; 15, Dengue virus; 16, Visna virus; 17, *Haemophilus influenzae*; 18, *Neisseria meningitidis*; 19, *Neisseria lactate*; 20, *Streptococcus pneumona*; 21, *Staphylococcus aureus*; 22, *Mycobacterium tuberculosis*; 23, *Klebsiella pneumoniae*; 24, Blank control (DW).

The analytical specificity of the MCDA-LFB assay was assessed using 21 non-EBV pathogens. As shown in **Figure 8**, two red lines (CL and TL) were visible on the LFBs when EBV standard plasmids and clinical sample were used as templates. In contrast, only one red line (CL) appeared on all non-EBV genomic DNA and blank control bands, indicating negative results. These findings were consistent with those from the VDR method, confirming that the EBV-MCDA-LFB assay accurately detected EBV pathogen with a 100% analytical specificity.

### Assessment of the feasibility of the EBV-MCDA-LFB assay in clinical samples

**Figure 9:**
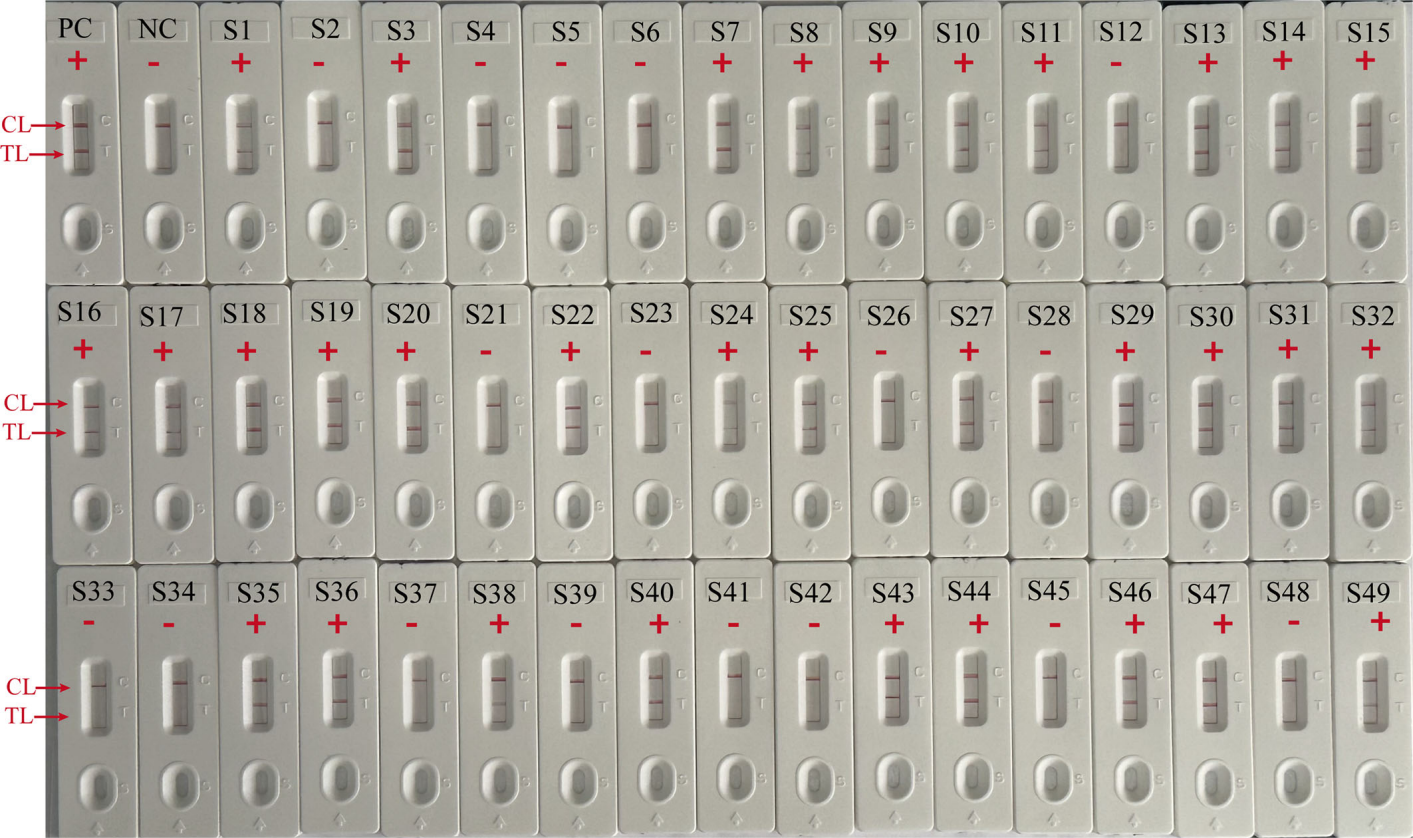
EBV-MCDA-LFB results on 49 suspected EBV infection plasma samples. S1 to S49 represents clinical samples 1 to 49. PC, positive control; NC, negative control; “ + “, positive; “-”, negative.

To further validate the feasibility of EBV-MCDA-LFB as a diagnostic tool for EBV, 49 plasma samples suspected of EBV infection were tested with EBV-MCDA-LFB and real-time qPCR assay simultaneously. Among them, 32 were classified as EBV-positive while 17 were EBV-negative according to the RT-qPCR results. EBV DNA were detected in 32 of 32 samples by EBV-MCDA-LFB technique (**Figure 9**), which were entirely consistent with the RT-qPCR outcomes (**Table 3**). The results indicated that the EBV-MCDA-LFB method, a simple, accurate, and rapid screening tool for EBV pathogens, has excellent potential to be widely used in clinical laboratory and basic research.

**Table 3:** Comparison of real-time qPCR and EBV-MCDA-LFB technique for the detection of EBV pathogen in clinical specimens.

Detection method	Clinical specimens	
	Positive	Negative
EBV-MCDA-LFB	32	17
Real-time qPCR	32	17

## Discussion

EBV, a highly prevalent virus worldwide, is closely associated with the occurrence and development of NPC. Unfortunately, the diagnosis of NPC is often delayed due to the hidden location and atypical symptoms, and most patients are already in advanced stages at the time of initial treatment, leading to a substantial decrease in the cure rate (Tan et al., 2020b). Effective population screening in high-incidence areas is essential to identify early-stage NPC and improve curative effect (Chen et al., 2019). EBV DNA is a pivotal biomarker for EBV-associated tumors and is clinically significance for screening, diagnosis, clinical staging, monitoring efficacy, and detecting microscopic residual lesions (Vasudevan and Yom, 2021). In a prospective study, analysis of EBV DNA in plasma samples demonstrated its effectiveness in identifying individuals at high risk for NPC, achieving a sensitivity and specificity of >97% (Chan et al., 2017). Traditional molecular detection methods, such as PCR and its derivatives (e.g., real-time qPCR, nested PCR.), have been regarded as sensitive, specific and reliable techniques for EBV DNA detection. However, the efficiency of PCR-based methods significantly varies due to differences in sample types, programming settings, and amplification equipment. In contrast to isothermal amplification methods [e.g. MCDA, loop-mediated isothermal amplification (LAMP)] as summarized in **Table 4**, PCR methods pose the disadvantages of high cost (relying on complex thermal cycling equipment for nucleic acid amplification), intricate manipulation (requiring skilled laboratory technicians for operation), and time-consuming protocols (with the entire detection process lasting 3-4 hours) (Vera-Sempere et al., 1998), limiting their widespread application in the PoC settings. (Wang et al., 2015; Cheng et al., 2023). Consequently, there is an imperative need to develop a rapid, accurate, and sensitive new technique for EBV detection and diagnosis.

**Table 4:** Comprehensive comparison of EBV-MCDA-LFB with other techniques for EBV detection.

Method	Sample type	Target gene	Limitation of detection	Processing time ^a^	Thermal cycling equipment ^b^	Reference
EBV-MCDA-LFB	plasma	BamHI-W fragment	7 copies/µl	60 min	N	–
LAMP	Serum, throat swab	BALF5	100 copies/tube	Approximately 70 min	N	(Iwata et al., 2006)
Competitive Q-PCR	peripheral blood, serum	EBNA-1	10 copies/reaction	NR	Y	(Stevens et al., 1999)
Conventional PCR	Serum	EBNA-1	1.69 x 10^5^ copies/ml (30 cycle) 1.69x10^3^ copies/ml (50 cycle)	Approximately 2h for 30 cycle PCR analyse 2.5h for 50 cycle PCR analyse	Y	(Hsiao et al., 2002)
Real-time qPCR	Serum	BNRF1	100 copies/ml	Approximately 100 min	Y	(Niesters et al., 2000)
Nested- PCR	paraffin-embedded tissues	EBNA-1	4 EBV genomes/cell	Approximately 3.5 h	Y	(Vera-Sempere et al., 1998)

a NR, not report;

b Y, yes; N, no

Herein, we report a novel technique for the specific detection and identification of plasma EBV based on MCDA and LFB. A distinct advantage of MCDA is the ability to rapidly and specifically amplify nucleic acids at constant temperatures (61-69°C), eliminating the need for expensive thermal cycling equipment. The amplification process can be completed within 40 minutes using simple equipment such as a metal bath, water bath pot, or a thermal cup (Wang et al., 2015; Cheng et al., 2023). Due to its abovementioned advantages, MCDA has found extensive utility in testing various pathogens, such as chlamydia trachomatis, monkeypox virus, and SARS-COV-2, achieving the remarkable limit of detection below 15 copies per reaction (Li et al., 2020; Chen et al., 2022; Zhou et al., 2023).

Currently, the commonly used analytical methods for nucleic acid amplification products, such as agarose gel electrophoresis, real-time turbidimetry, or colorimetry, cannot be widely used in resource-limited areas due to the requirement for expensive instruments, specialized detection reagents, and skilled technical support. In this study, we used nanoparticle-based LFB to analyze MCDA amplification results. Nanoparticles are the most widely used nanomaterials for biosensors due to their attributes, which include high adsorption, high specific surface area, excellent biocompatibility, favorable surface effect, and unique optical properties (Aldewachi et al., 2017; Huang et al., 2019). LFB can directly monitor biotin/FITC-labeled target amplicons, which is simple to operate and obtain visual results within 2 minutes without expensive reagents and complex instruments. It is rapid, sensitive, and economical for *in-situ* diagnosis. The whole process of MCDA-LFB method for EBV DNA detection, including sample preparation (15 minutes), MCDA (40 minutes), and result reading (2 minutes) can be completed within 60 minutes. This approach eliminates the reliance on complex thermal cycling equipment, simplifies the pathogen detection process, and fulfills the demand for PoC detection and rapid in-field testing (Wang et al., 2017; Jiao et al., 2019; Cheng et al., 2023).

In this study, we investigated the optimal reaction temperature for the EBV-MCDA-LFB assay. Our results revealed that the MCDA displayed maximal amplification efficiency at 65°C, surpassing the performance at alternative reaction temperatures. Furthermore, we determined a LoD concentration of 7 copies per microliter by 10-fold serial dilution of the EBV standard plasmids. This concentration level can be reliably detected within 40 minutes of continuous amplification. To evaluate the specificity of the MCDA-LFB method, we thoroughly examined 21 diverse bacteria and viruses. The EBV-MCDA-LFB test demonstrated superb specificity by producing positive results only for the EBV pathogen and negative results for non-EBV pathogen. Additionally, we validated the feasibility of our approach for detecting EBV in plasma by comparing it with the RT-qPCR method. Notably, our outcomes exhibited 100% concordance between positive and negative diagnoses of EBV DNA. The findings imply that the EBV-MCDA-LFB method, which we developed, is a rapid, highly sensitive, and specific technique for detecting EBV. It shows potential for application in clinical and laboratory settings.

The EBV-MCDA-LFB technique also possesses drawbacks and limitations that require consideration. It should be noted that the MCDA process yields a vast quantity of amplification products. Upon unsealing the reaction container, there is a risk of releasing substantial quantities of target DNA amplicons into the surrounding environment, leading to potential contamination and false positive results. Therefore, rigorous measures are crucial to minimize contamination and improve specificity. Maintaining strict spatial separation between sample preparation and amplification, along with regular glove changes will help to reduce the risk of cross contamination. When monitoring amplified products using LFB, aspirating smaller volumes of reaction products may mitigate potential viral aerosol contamination to some extent. Our research suggests that employing only 0.5 µl of amplified products is sufficient for effective detection by the LFB assay. Additionally, applying 70% ethanol and sodium hypochlorite solution promptly to the workspace after analyzing the results can assist in mitigating DNA contamination (Cheng et al., 2023). Addressing the challenge of minimizing such contamination remains a critical aspect for future studies in this field.

## Conclusion

In conclusion, we have developed a rapid and straightforward visualization method for detecting EBV DNA in plasma utilizing MCDA-LFB assay. The procedure can be completed within 60 minutes without the need for expensive equipment and specialized reagents. The LoD level of this method was determined to be 7 copies per microliter for EBV recombinant plasmid, and there was no observed cross-reactivity with other pathogen strains. This method holds significant clinical application prospects, particularly in the early screening of asymptomatic NPC using plasma samples.

## Data availability statement

The original contributions presented in the study are included in the article/supplementary materials. Further inquiries can be directed to the corresponding authors.

## Ethics statement

The studies involving humans were approved by The Human Ethics Committee of the Children’s Hospital Capital Institute of Pediatrics. The studies were conducted in accordance with the local legislation and institutional requirements. Written informed consent for participation in this study was provided by the participants’ legal guardians/next of kin.

## Author contributions

XJ: Investigation, Validation, Writing – original draft. JZ: Methodology, Validation, Writing – review & editing. FX: Methodology, Writing – review & editing. XH: Investigation, Methodology, Writing – review & editing. WQH: Methodology, Writing – original draft. WH: Writing – review & editing. YK: Writing – original draft. WY: Writing – original draft. JJ: Writing – original draft. YQ: Writing – original draft. YW: Methodology, Project administration, Software, Supervision, Writing – review & editing. JT: Funding acquisition, Resources, Validation, Writing – review & editing.
